# A tandem CBM25 domain of α-amylase from *Microbacterium aurum* as potential tool for targeting proteins to starch granules during starch biosynthesis

**DOI:** 10.1186/s12896-017-0406-x

**Published:** 2017-12-04

**Authors:** Xing-Feng Huang, Farhad Nazarian, Jean-Paul Vincken, Richard G. F. Visser, Luisa M. Trindade

**Affiliations:** 1 Wageningen University and Research, Plant Breeding, P.O. Box 386, 6700 AJ Wageningen, The Netherlands; 2 Present address: Department of Chemical and Biological Engineering, Colorado State University, Campus delivery 1370, Fort Collins, CO 80523 USA; 3 Present address: Agronomy and plant breeding group, Faculty of Agriculture, University of Lorestan, P.O.Box 465, Khorramabad, Iran; 4 Present address: Laboratory of Food Chemistry, Wageningen University, P.O. Box 8129, 6700 EV Wageningen, The Netherlands

**Keywords:** Starch, Transgenic potato, Starch binding domain, CBM20, CBM25

## Abstract

**Background:**

Starch-binding domains from carbohydrate binding module family 20 have been used as a tool for starch engineering. Previous studies showed that expression of starch binding domain fusion proteins *in planta* resulted in modified starch granule structures and physicochemical properties. However, although 13 carbohydrate binding module families have been reported to contain starch-binding domains, only starch-binding domains from carbohydrate binding module family 20 have been well studied and introduced into plants successfully.

In this study, two fragments, the tandem CBM25 domain and the tandem CBM25 with multiple fibronectin type III (FN3) domains of the α-amylase enzyme from *Microbacterium aurum*, were expressed in the tubers of a wild type potato cultivar (cv. Kardal) and an amylose-free (*amf*) potato mutant.

**Results:**

The (CBM25)_2_ and FN3 protein were successfully accumulated in the starch granules of both Kardal and *amf* transformants. The accumulation of (CBM25)_2_ protein did not result in starch morphological alterations in Kardal but gave rise to rough starch granules in *amf*, while the FN3 resulted in morphological changes of starch granules (helical starch granules in Kardal and rough surface granules in *amf*) but only at a very low frequency. The starches of the different transformants did not show significant differences in starch size distribution, apparent amylose content, and physico-chemical properties in comparison to that of untransformed controls.

**Conclusion:**

These results suggest that the starch-binding domains from carbohydrate binding module family 25 can be used as a novel tool for targeting proteins to starch granules during starch biosynthesis without side-effects on starch morphology, composition and properties.

**Electronic supplementary material:**

The online version of this article (10.1186/s12896-017-0406-x) contains supplementary material, which is available to authorized users.

## Background

Starch is the most important and abundant storage carbohydrate in plants and exists in the form of discrete granules in higher plants such as wheat, rice, maize, potato, and pea. Starch granules are mainly composed of two α-glucan polymers, a mostly linear amylose (20-30%) and a highly branched amylopectin (70-80%) [1]. Starches are used in food and many other industrial applications, but for most of the industrial applications, starch normally needs to be modified to overcome certain physico-chemical limitations such as: low shear and thermal resistance, and a high tendency towards retrogradation [2]. Although starch modification can be conducted by chemical and/or physical treatments, these treatments are time and money consuming or they generate environmental pollution. Starches with novel properties can be produced by modification of the starch biosynthetic pathway. Nowadays, using modern molecular tools it becomes possible to generate plants which can produce so-called “tailor-made” starches that can be used directly for specific industrial uses without extra post-harvest treatments. For example, starches with altered amylose/amylopecin ratio have been successfully achieved by controlling the expression levels of genes which are involved in starch biosynthesis [3, 4].

Microbial extracellular hydrolytic enzymes that catalyze the degradation of insoluble polysaccharides, such as glycogen, starch, and cellulose, typically possess a modular architecture referred to as carbohydrate-binding modules (CBMs) which target cognate catalytic modules to specific polysaccharide structure [5, 6]. To date, various types of CBMs have been recognized and classified into 59 different CBM families based on their amino acid similarity [7]. CBMs which show affinity to starch granules are called Starch-Binding Domains (SBDs). Currently, there are 13 CBM families, named CBM20, CBM21, CBM25, CBM26, CBM34, CBM41, CBM45, CBM48, CBM53, CBM58, CBM68, CBM69, and CBM74 which have been reported to contain SBDs [8]. SBDs are about 100 amino acids long and have well conserved amino acid sequences among different families [9, 10]. It is known that SBDs play the following three roles: they allow the interaction between the insoluble substrate and the enzyme in solution; they help the catalytic domain of enzymes to catch the substrate; and in some cases they have a disruptive function on the starch granule surface [11].

Until now, only SBDs from CBM family 20 have been well studied [5] and introduced into plants successfully [12, 13]. For instance, using the SBD as an anchoring tool, an active luciferase-SBD fusion protein was accumulated inside starch granules, whereas luciferase alone could not [14]. Previous studies also showed that expression of starch binding domain fusion proteins *in planta* resulted in altered starch granule structures and properties [15–17]. For example, the expression of an engineered granule-bound *Escherichia coli* glycogen branching enzyme in potato resulted in severe morphological changes in starch granules [15]. Expression of an amylosucrase gene in potato results in larger starch granules with novel properties [16]. Moreover, expression of an engineered laforin (a human enzyme composed of a carbohydrate-binding module and a dual-specificity phosphatase domain) in potato resulted in significantly higher phosphate content of starch [18].

It is still unknown whether the SBDs of other CBM families can be used to target proteins to starch granules and whether they have other different biochemical properties in comparison to CBM20s. A multi-domain α-amylase enzyme composed of a catalytic α-amylase domain, multiple fibronectin type III domains, and a tandem carbohydrate binding domain belonging to the CBM family 25, was isolated from *Microbacterium aurum*. This α-amylase enzyme is responsible for the formation of numerous small pores in starch granules when it was incubated with potato starch [19]. The tandem CBM25 domain and the repeated fibronectin III domain are responsible for α-amylase domain catching and working on starch granules and deletion of the fibronectin type III and the tandem CBM25 resulted in loss of α-amylase function [19].

In this study, the effects of the tandem CBM25 domain on starch granules were studied and the potentiality of using this tandem CBM25 domain as tool for anchoring proteins into starch granules during starch biosynthesis was assessed.

## Results

### No visible phenotype in plant architecture and tuber morphology

**Fig. 1:**
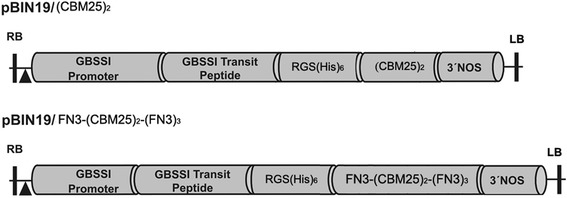
Schematic representation of the full amino acid sequences of the two different constructs used in this study for potato transformation. Recombinant genes are driven by potato granule-bound starch synthase I (GBSSI) promoter and the amyloplast targeting is ensured by potato GBSSI transit peptide

Two constructs (Fig. 1), which are referred to as pBIN19/(CBM25)_2_ (the tandem CBM25 domain sequence) and pBIN19/FN3 (tandem CBM25 with multiple fibronectin type III domains) were transformed into wild type potato (Kardal) and amylose-free potato (*amf*), respectively. Thirty independent kanamycin-resistant transformant clones from each transformation were transferred to the greenhouse for tuberization and further analysis. During the growth in the greenhouse, the morphology of transgenic plants did not show detectable differences when compared with that of untransformed control plants. The morphology of tubers, the tuber yield, and the starch yield of all transformants were comparable with that of untransformed controls (data not shown).

### mRNA transcript analysis of (CBM25)_2_ and FN3 transformants

**Fig. 2:**
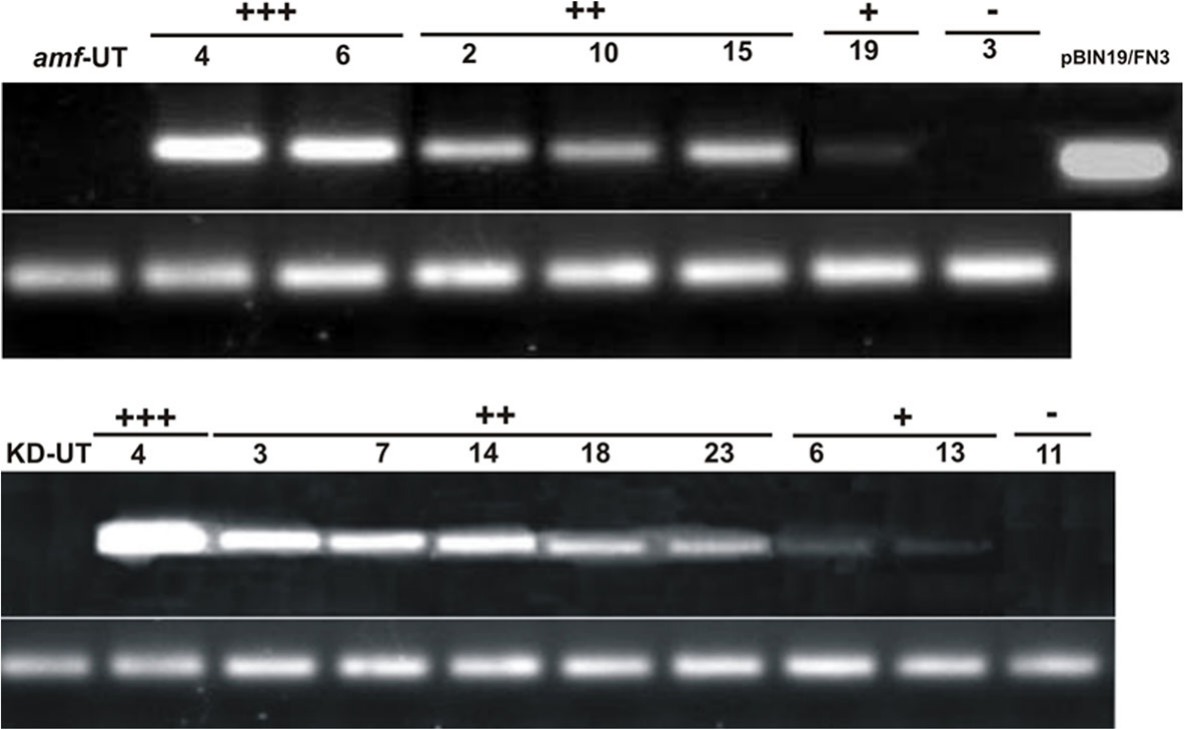
Semi-quantitative RT-PCR with specific primer pairs for KD-FN3 and *amf*-FN3 transformant series. The lower panel shows PCR product of the internal control gene Ubiquitin (Ubi3)

Expression of the two gene fragments, (CBM25)_2_ and FN3, in potato tubers was determined by semi-quantitative RT-PCR. Results showed that tubers of transgenic plants possess the mRNA transcripts of the expressed fusion proteins (Fig. 2). Moreover, the intensity of semi-qRT-PCR products varied in different transformants. Based on the band intensity of the PCR products, the transformants were divided into four different classes (−, +, ++, and +++ representing transformants with no, low, medium and high levels of mRNA, respectively).

### (CBM25)_2_ and FN3 proteins are present in starch granules

The expression of (CBM25)_2_ and FN3 proteins in both Kardal and *amf* potato starches was analyzed by Western dot blot. An N-terminally engineered RGS(His)_6_-tag allows the detection and classification of the accumulation of the His-tagged fusion proteins in starch granules from transformants expressing different constructs. The Western dot blot results showed that the His-tag fusion proteins can be detected in (CBM25)_2_ and FN3 transformant series both in Kardal and *amf* backgrounds (data not shown).

As predicted, the molecular masses of the His-tagged fusion proteins were 22,954 Da and 59,283 Da for (CBM25)_2_ and FN3 respectively. Starch granule bound proteins were extracted from different transformants and were analyzed with SDS-PAGE and subsequent Western blotting immuno-detection with anti-RGS(His)_6_ antibodies. As shown in Additional file 1: Figure S1, bands with the right size were present in starch granules of (CBM25)_2_ and FN3 transformed plants and bands were undetectable in both untransformed control Kardal and *amf* starches. These results indicate that (CBM25)_2_ fused with poly-His-tag and FN3 fused with poly-His-tag fusion proteins have been targeted to the starch granules successfully.

### Starch granule morphology remains unaltered in most transformants

The (CBM25)_2_ and FN3 proteins were accumulated in starch granules of transformants in both Kardal and *amf* backgrounds. In order to investigate whether the accumulation of fusion proteins in starch granules affect the starch granule morphology, starch granules of each transformant were investigated by both light microscopy and electron scanning microscopy (SEM).

**Fig. 3:**
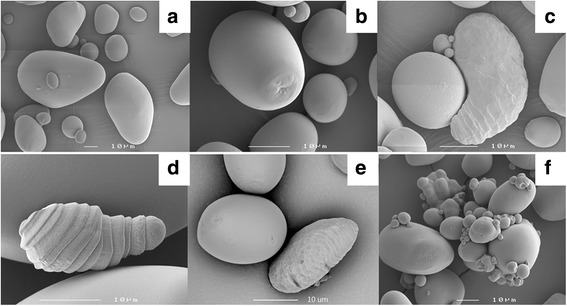
SEM analyses of starch granules from KD-UT (**a**) and *amf* (**b**) in comparison with that of the different selected transformants: *amf*-2CBM25-29 (**c**), KD-FN3-07 (**d**), *amf-*FN3-10 (**e**), and *amf*-FN3-15 (**f**). The scale bar in each picture indicates different magnifications

Light micrographs showed that the accumulation of (CBM25)_2_ and FN3 proteins did not affect starch granule morphology in Kardal background. Most of the transformants did not show any starch granule morphological alteration in KD-(CBM25)_2_ and KD-FN3 transformant series in comparison with that of untransformed control. However, 2 out of 30 KD-FN3 transformants, KD-FN3-04 and KD-FN3-07, showed an altered starch morphological phenotype. Starch granules from these transformed plants showed a helical shell-shaped structure at a very low frequency (less than 1%) which was observed when starch was stained with iodine. Further investigations with SEM revealed the feature of these structures in more detail (Fig. 3d). As shown in Fig. 3, the starch granules from control samples (Fig. 3a) have smooth surfaces while helical shell-shaped starch granule was detected in KD-FN3-07 transformant (Fig. 3d). Furthermore, it is worth to note that the two transformants KD-FN3-04 and KD-FN3-07, which have some altered starch granules, belong to high level and medium level in the mRNA transcript analysis of FN3 (Fig. 2).

In *amf* background, the expression of both proteins did not result in severe alterations in starch granule morphology. The light micrographs showed that the *amf*-(CBM25)_2_ and *amf*-FN3 transformants did not show starch morphological alterations (data not shown). However, SEM microscopy revealed that the surface of starch granules from both *amf*-2CBM25 and *amf*-FN3 transformants were rough, especially in the higher expressors such as *amf*-2CBM25-29 and *amf*-FN3-10 (Fig. 3c and e). There were 5 out of 32 transformants in *amf*-(CBM25)_2_ and 13 out of 18 transformants in *amf*-FN3 that showed rough surface granules albeit at a low percentage. Moreover, there was one transformant (*amf*-FN3-15) that showed amalgamated starch granules (Fig. 3f).

To investigate the frequency of starch granules with altered morphology, the number of altered granules was scored by counting a population of 300 starch granules in triplicate for selected transformants which have the highest fusion protein accumulation level. The results from these quantification analyses showed that the helical starch granules were present at a very low percentage (less than 1%) in KD-FN3-04 and KD-FN3-07 transformants. The starch granules with rough surface in *amf*-(CBM25)_2_ and *amf*-FN3 transformants were also present at a low frequency (about 5%) in the highest expressors (*amf*-(CBM25)_2_-29 and *amf*-FN3-04).

### Starch granule size distribution, gelatinization behavior, and amylose content remain unchanged

The impact of (CBM25)_2_ and FN3 protein accumulation in starch granules on starch physico-chemical properties, such as apparent amylose content, mean granule size and granule size distribution, and starch granule melting behavior (Tonset and ΔH), was measured. These results are summarized in Table 1. As shown in Table 1, no significant differences were observed between the starches of *amf*-(CBM25)_2_, KD-FN3, and *amf*-FN3 transformants and their corresponding controls.

**Table 1:** Summary of different starch characteristics in relationship with class of semi-qRT-PCR. Starch apparent amylose content (%AM), median granule size(d_50_), and starch gelatinization temperature are shown. %AM, d_50,_ ΔH, and T onset data are average of three independent measurements with the standard deviation

	semi-qPCR class	AM(%)	d50(μm)	ΔH (kJ/g)	Tonset(°C)
KD-UT	–	22.5 ± 0.5	22,2 ± 0.3	13.6 ± 0.4	64.7 ± 0.6
KD-FN3-11	0	21.3 ± 0.6	17.3 ± 0.2	12.2 ± 0.3	65.5 ± 0.4
KD-FN3-13	1	21.0 ± 1.3	19.5 ± 0.4	12.9 ± 0.2	65.0 ± 0.3
KD-FN3-03	2	22.2 ± 0.9	20.0 ± 0.2	13.6 ± 0.2	65.3 ± 0.4
KD-FN3-07	2	21.8 ± 1.5	18.2 ± 0.3	11.9 ± 0.5	66.7 ± 0.6
KD-FN3-04	3	21.3 ± 0.6	19.0 ± 0.1	12.5 ± 0.3	66.4 ± 0.4
amf-UT	–	3.3 ± 0.2	10.5 ± 0.2	13.7 ± 0.4	70.2 ± 0.5
amf-FN3-03	0	3.9 ± 0.3	8.1 ± 0.1	12.3 ± 0.4	69.5 ± 0.3
amf-FN3-19	1	3.4 ± 0.3	9.3 ± 0.2	14.1 ± 0.3	69.0 ± 0.2
amf-FN3-10	2	3.6 ± 0.2	7.0 ± 0.1	10.7 ± 0.5	69.2 ± 0.2
amf-FN3-15	2	3.9 ± 0.3	8.6 ± 0.0	13.4 ± 0.2	69.2 ± 0.1
amf-FN3-06	3	3.6 ± 0.4	8.7 ± 0.1	12.1 ± 0.3	69.6 ± 0.3
amf-UT	–	2.9 ± 0.6	9.1 ± 0.1	16.0 ± 0.3	73.3 ± 0.3
amf-2CBM25-06	0	3.8 ± 0.3	7.6 ± 0.0	16.5 ± 0.1	73.5 ± 0.4
amf-2CBM25-23	1	3.4 ± 0.1	9.1 ± 0.1	16.9 ± 0.4	72.7 ± 0.4
amf-2CBM25-12	2	3.0 ± 0.6	8.2 ± 0.1	15.8 ± 0.3	73.6 ± 0.5
amf-2CBM25-29	3	3.2 ± 0.5	8.2 ± 0.0	16.8 ± 0.5	73.3 ± 0.4

## Discussion and conclusions

In this study, the tandem CBM25 sequence and the tandem CBM25 with repeated fibronectin type III domain sequence from *M. aurum* were expressed in amylose containing and amylose-free potatoes, respectively, to test the potentiality of the tandem CBM25 domain as a tool to target proteins into the starch granules during starch biosynthesis.

The tandem CBM25 domain has been successfully incorporated into potato starch granules during starch biosynthesis in both Kardal and in *amf* genetic backgrounds in this study (Fig. 3). Starch-binding domains from CBM family 20 have been well studied and previous studies found the SBD from CBM20 accumulated inside the starch granules in transgenic plants without any detectable side effects on starch properties [14]. Another study showed that both a single SBD and an engineered repeat SBD of the CGTase from *Thermoanaerobacterium thermosulfurigenes* have been incorporated in the chloroplast of *Arabidopsis thaliana* wild type and the starch excess mutant (*sex 1-1*) [12]. These above-mentioned studies demonstrated that the SBDs can be used as tools for targeting proteins into starch granules. Furthermore, the SBD from CBM20 has been successfully used as tool to introduce enzymes or enzyme domains, which do not have affinity for starch granules, into starch granules to modify starch structure and physico-chemical properties [15, 16]. This technique provides a promising future for starch modification *in planta* by using SBDs. In this study, the expression of the tandem CBM25 did not affect starch composition (apparent amylose content) and starch physico-chemical properties (Table 1). This is in agreement with results of previous studies except for the size distribution of starch granules which did not change in this study as was the case when tandem CBM20 SBDs were expressed in potato tubers or Arabidopsis leaves [12, 13].

The accumulation of the tandem CBM25 domain in starch granules neither lead to severe starch morphology alterations nor affected the starch granule size distribution, but there are some altered starch granules at a very low frequency were observed in this study. For instance, the helical starch granules were only observed in 2 out of 30 KD-FN3 transformant plants at a very low frequency (less than 1%) and the rough surface granules (approximately 5%) showed in both *amf*-(CBM25)_2_ and *amf*-FN3 transformants. The rough surface granule phenotype in this study and the amalgamated starch granules which were only observed in *amf*-FN3-15 are similar to the starch morphological phenotypes which were reported in the double CBM20 tranformants in *amf* background [13]. These starch morphological alterations suggest that the (CBM25)_2_ and FN3 protein might interfere with the starch granule assembling process to some degree to give rise to starch morphological alterations, though at a very low rate.

In the 13 CBM families which contain SBDs, the structure and property of CBM20 and CBM25 have been thoroughly characterized [20–22]. Although both CBM20 and CBM25 have affinity for starch granules, they do not interact with α-glucans in the same way. First of all, the SBD from CBM20 has two binding sites with distinct affinities for malto-oligosaccharides, whereas the CBM25 only have one binding site. Furthermore, the CBM20 only binds the non-reducing termini of two parallel side chains of amylopectin [23], but CBM25 binds the linear single α-glucan chain in both amylose single helix and amylopectin double helix [20]. Taken the structure and mechanism of CBMs binding to α-glucans into consideration, the interaction of tandem CBM25 used in this study with α-glucans in starch granules has similarities with the single CBM20 which contains two binding sites. Moreover, because the CBM25 binds single α-glucan chains, it likely binds with both amylose and amylopectin during starch biosynthesis and therefore can be present in both crystalline and non-crystalline regions of starch.

The absence of amalgamated granules may be due to the fact that the double CBM25 has less binding sites than the double CBM20. It was proposed by Nazarian and co-workers [24] that the tandem CBM20 can cross-link different starch granule nucleation sites and lead to the amalgamated granules which are observed in the high 2CBM20 expressers. Since the tandem CBM25 only has two binding sites in total, the mode of action would be similar to the single SBD (2 binding sites). It is therefore not surprising that most of the transformants did not show amalgamated starch granules, as observed in the double CBM20 transformants in both Kardal and *amf* backgrounds [13]. However, there is one transformant *amf*-FN3-15 with similar amalgamated starch granule phenotype suggesting that the accumulation level of (CBM25)_2_ could lower than (CBM20)_2_, although this cannot be confirmed by the comparison of these two proteins accumulation level due to two different antibodies were used for Western Blotting detection. Moreover, the helical granules which were observed in two KD-FN3 transformants have never been observed before in any of the transgenic potato starches. Since the mRNA expression level of FN3 in both transformants is quite high comparing with that of other transformants, the expression of FN3 could affect genes involved in starch assembling process and give rise to these helical starches, though at a very low rate. However, this needs to be investigated further.

Since the incorporation of the tandem CBM25 in starch does not affect physico-chemical properties of starch granules, in particular gelatinization of starch, and it is supposed to deposit in all the regions of the starch granule, the tandem CBM25 domain can be a better tool than CBM20 to anchor proteins into starch granules in order to maximize the potential for the effector proteins. This study shows that CBM25 is a very good (alternative) tool to anchor effector proteins into starch granules for starch bioengineering.

## Methods

### Construction of different expression vectors

Two DNA fragments, the (CBM25)_2_ tandem sequence and the (CBM25)_2_ with repeated fibronectin type III domain sequence of the α-amylase from *M. aurum*., were cloned in frame to the GBSSI promoter and its transit peptide sequence with a RGS(His)_6_-tag to facilitate antibody detection. The two constructs, named pBIN19/(CBM25)_2_ and pBIN19/FN3 were made in pBIN19 expression binary vector. These constructs were made to express (CBM25)_2_ and FN3-(CBM25)_2_-3FN3 fusion proteins in potato plants. To obtain the pBIN19/(CBM25)_2_ plasmid (Fig. 1), a (CBM25)_2_-encoding fragment was amplified by PCR using the primers 5′-ACCCATGGCAGAGAAGCAGGCGTCGACGTC -3′ and 5′-GGGATCCGGCATCACGGGTCCTTCACCGACAC -3′, that comprised the *Nco*I and *BamH*I sites at their 5′ ends, respectively. *Microbacterium aurum* DNA was used as a template for PCR amplification. This amplified fragment was used to replace the SBD2 fragment (also *Nco*I-*BamH*I) in the pUC19/SBD2 plasmid [13], giving rise to the pUC19/(CBM25)_2_ plasmid. Similarly, the other fragment was cloned into the same pUC19/SBD2 plasmid with the primers 5′-AGCCCATGGTGCGTGCACCGAC -3′ and 5′-ATCGGATCCTTACGCCGAAGTACCTGCC -3′, for FN3-(CBM25)_2_-3FN3 amplifications. The 5′ end of the coding regions in all constructs carried a RGS(His)_6_ tag to facilitate protein detection and purification. All constructs in pUC19 were later digested with *Hpa*I-*BamH*I and cloned into the same position in pBIN19 vector containing potato granule-bound starch synthase I (GBSSI) promoter and its transit peptide for tuber expression and amyloplast targeting and a NOS terminator, respectively. The pBIN19/(CBM25)_2_ and pBIN19/FN3 were assembled from the following fragments: (1) GBSSI promoter and transit peptide, (2) RGS(His)_6_-(CBM25)_2_ or RGS(His)_6_-FN3-(CBM25)_2_-(FN3)_3_, and (3) a NOS terminator. The predicted molecular masses of the RGS(His)_6_ containing proteins were 22,954 Da and 59,283 Da for RGS(His)_6_-(CBM25)_2_ and RGS(His)_6_-FN3 respectively, excluding the transit peptide. The accuracy of all constructs were controlled by sequence analysis.

### Potato transformation

*Agrobacterium tumefaciens* strains LBA4404 harboring the expression cassette constructs (Fig. 1) was used for transformation according to the three-way method by Visser et al. [25]. Murashige and Skoog medium (MS30) containing100 mg/L kanamycin were used in tissue culture experiments [26], Amylose-free potato mutant (*amf*) and amylose-containing potato plants (wild-type) were used for transformations. Two constructs (Fig. 1), which are referred to as pBIN19/(CBM25)_2_ and pBIN19/FN3 were transformed in Kardal and *amf*, respectively. Thirty kanamycin-resistant individual transformant clones from each transformation were transferred to the greenhouse for tuberization and further analysis. (CBM25)_2_ refers to the (CBM25)_2_ domain; FN3 refers to (CBM25)_2_ with repeated fibronectin type III domain. KD-UT and *amf*-UT refers to two untransformed controls respectively, and xx stands for the clone number. Plantlets grown on MS30 selection medium were harvested, multiplied and 5 plantlets of each clone was subsequently grown in pots in the greenhouse to generate tubers.

### Starch isolation

Starches were isolated according to the method by Huang et al. [15]. Briefly, the tubers from each individual transformant were peeled, mixed and ground. Collected juices were settled at 4 °C for at least 3 h. Then, the settled starch was washed and filtered through a Whatman No. 1 filter paper. The starch samples wereair-dried and collected at room temperature.

### Western blot analysis

The amount of RGS(His)_6_-tag fusion proteins accumulated in the transgenic starch granules was measured by Western dot blot analysis. For determining the amount of fusion proteins, 50 mg of (transgenic/control) starches were boiled for 5 min with 1 mL of 2× SDS sample buffer containing 5% β-mercaptoethanol (*v*/v). After cooling to room temperature, equal amount of the supernatants (500 μL) was applied to a Hybond ECL transfer membrane (Amersham, UK) using the S&S manifold-I dot blot system (Schleicher & Schuell, Keene, NH, USA). To detect expression of recombinant His-tagged proteins, a monoclonal anti-RGS(His)_6_-tag antibody was obtained from Qiagen (91,974 Courtaboeuf Cedex). Protein fractions were electroblotted onto nitrocellulose membrane. Probing and detection of Western-dot blots were performed as described by Ji et al. [14]..The SDS-PAGE was carried out according to Huang et al. [15] and the proteins were detected using anti-RGS(His)_6_-tag (Qiagen) according to instruction of manufacturer.

### Semi-quantitative RT-PCR analysis

Relative expression of mRNA was determined by Semi-quantitative RT-PCR analysis as described by Nazarian et al. [27]. The ubiquitin gene (L22576) was used as an internal control [28].

### Analysis of physiochemical properties of starches

A combination of light microscopy (LM; Axiophot, Germany) and scanning electron microscopy (SEM; JEOL 6300F, Japan) were used to investigate the granule morphology of starch granules as described by Huang et al. [15].

The apparent amylose content of starch granules were measured using the method by Hovenkamp-Hermelink et al. [29]. Theonset temperature (Tonset) of starch granule melting and granule size distributions were measured as described by Huang et al. [15].

### Statistical analyses

One-way analysis of variance (ANOVA) was carried out to compare the statistical difference in starch apparent amylose content (%AM), median granule size(d50), and starch gelatinization temperature of the transformants and controls. In Table 1, each value represents the average of three independent measurements and the standard deviation.

## Additional file

Additional file 1: Figure S1. Western blot analysis of the highest expressor of 2CBM25 and FN3 transformants. Total proteins were extracted from starch granules of controls (*amf* and Kardal) and one representative of each series of lines. Lane 1, 2, 3, 4, 5 represent *amf*-2CBM25-29, *amf* control, KD-FN3-04, amf-FN3-15, and Kardal control, respectively. Western blot analysis was performed by using an anti-RGS(His)_6_ antibody. The molecular mass ladder is indicated. (TIFF 14274 kb)
