# Supplementary figures and images for: A tandem CBM25 domain of α-amylase from Microbacterium aurum as potential tool for targeting proteins to starch granules during starch biosynthesis
Source: BMC Biotechnol. 2017 Dec 4;17:86. doi: 10.1186/s12896-017-0406-x (PMC5715617; doi:10.1186/s12896-017-0406-x)

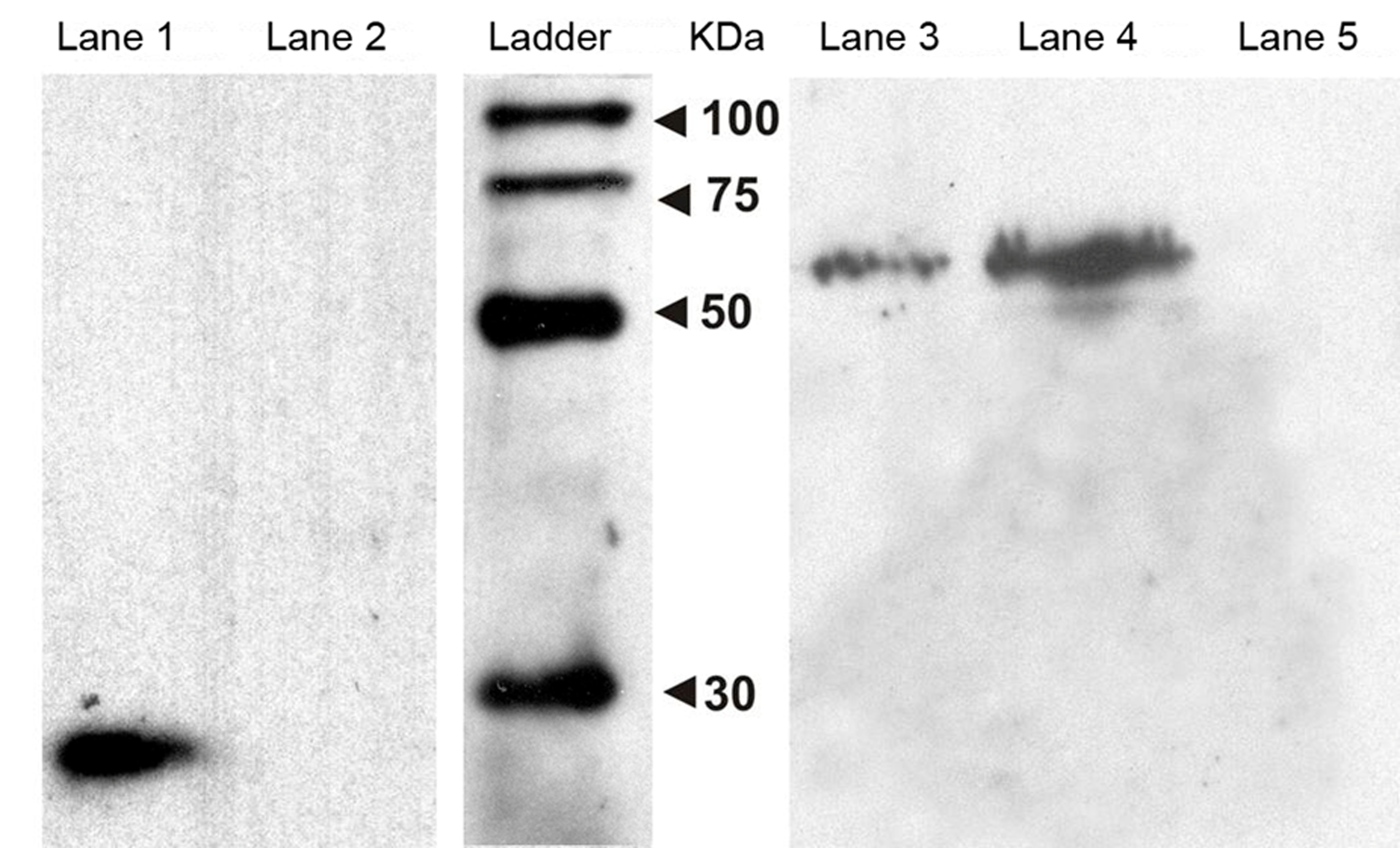

Supplement: Additional file 1: Figure S1. — Western blot analysis of the highest expressor of 2CBM25 and FN3 transformants. Total proteins were extracted from starch granules of controls (amf and Kardal) and one representative of each series of lines. Lane 1, 2, 3, 4, 5 represent amf-2CBM25-29, amf control, KD-FN3-04, amf-FN3-15, and Kardal control, respectively. Western blot analysis was performed by using an anti-RGS(His)6 antibody. The molecular mass ladder is indicated. (TIFF 14274 kb) [file 12896_2017_406_MOESM1_ESM.tif]
